# Supplementary figures and images for: Continued T12 transmission and shared antibiotic resistance during 2018–2023 Vibrio cholerae outbreaks in Cameroon
Source: PLOS Glob Public Health. 2025 Feb 24;5(2):e0003763. doi: 10.1371/journal.pgph.0003763 (PMC11849833; doi:10.1371/journal.pgph.0003763)

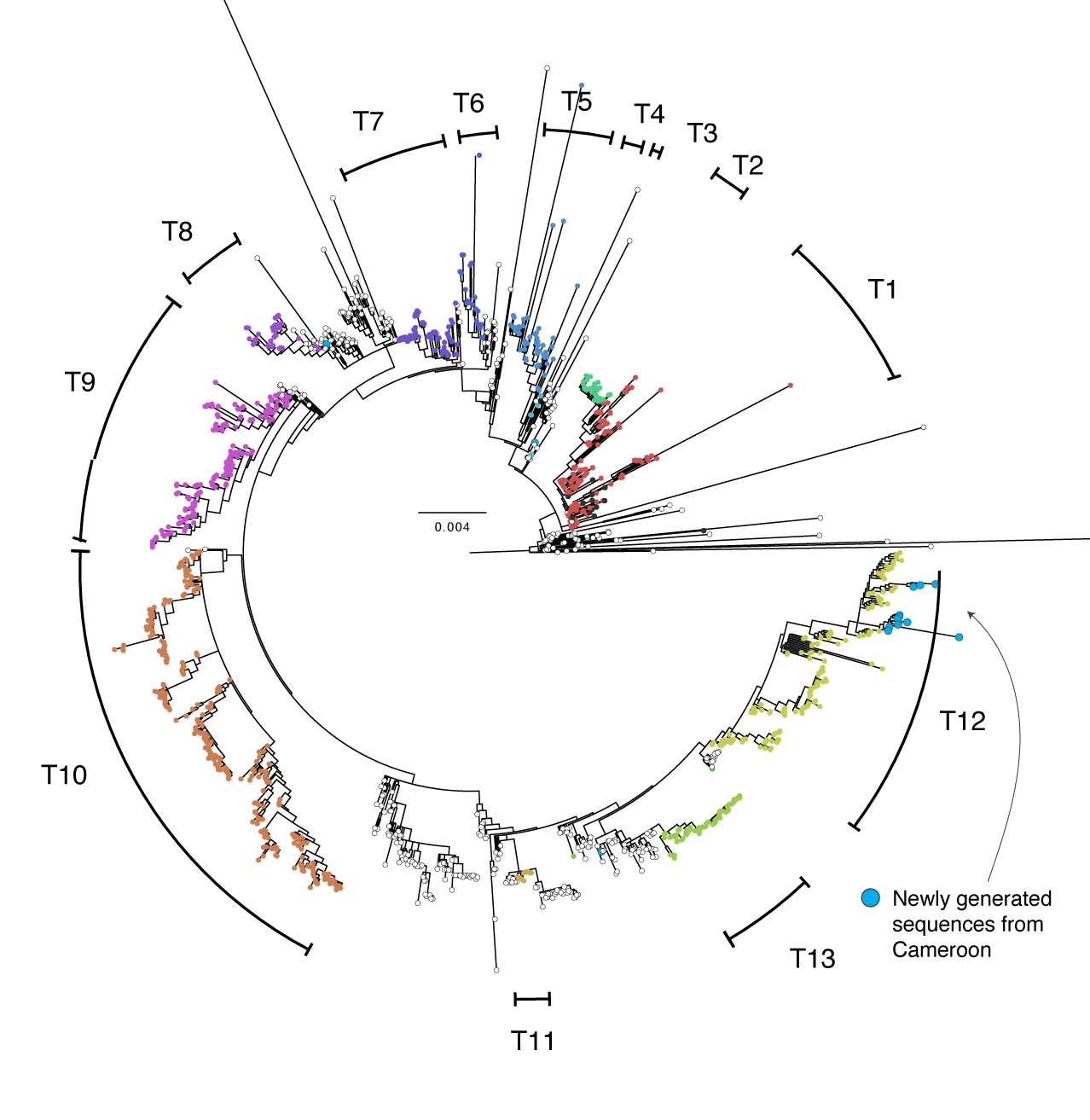

Supplement: S1 Fig — Maximum likelihood reconstruction of 13 newly generated sequences from Cameroon plus 1383 previously published background sequences. Tips are colored by introduction event and newly generated sequences are indicated in blue. (TIF) [file pgph.0003763.s006.tif]
